# Activation of Both the Calpain and Ubiquitin-Proteasome Systems Contributes to Septic Cardiomyopathy through Dystrophin Loss/Disruption and mTOR Inhibition

**DOI:** 10.1371/journal.pone.0166839

**Published:** 2016-11-23

**Authors:** Ana Caroline Silva Freitas, Maria Jose Figueiredo, Erica Carolina Campos, Danilo Figueiredo Soave, Simone Gusmao Ramos, Herbert B. Tanowitz, Mara Rúbia N. Celes

**Affiliations:** 1 Department of Pathology, Faculty of Medicine of Ribeirao Preto, University of Sao Paulo, Sao Paulo, Brazil; 2 Department of Physiotherapy, Faculty of Physical Education, Federal University of Uberlandia, Minas Gerais, Brazil; 3 Department of Histology, Embryology and Cellular Biology, Federal University of Goias, Goias, Brazil; 4 Departments of Pathology and medicine, Albert Einstein College of Medicine, Yeshiva University, Bronx, New York, United States of America; 5 Institute of Tropical Pathology and Public Health, Federal University of Goias, Goias, Brazil; Western University, CANADA

## Abstract

Cardiac dysfunction caused by the impairment of myocardial contractility has been recognized as an important factor contributing to the high mortality in sepsis. Calpain activation in the heart takes place in response to increased intracellular calcium influx resulting in proteolysis of structural and contractile proteins with subsequent myocardial dysfunction. The purpose of the present study was to test the hypothesis that increased levels of calpain in the septic heart leads to disruption of structural and contractile proteins and that administration of calpain inhibitor-1 (N-acetyl-leucinyl-leucinyl-norleucinal (ALLN)) after sepsis induced by cecal ligation and puncture prevents cardiac protein degradation. We also tested the hypothesis that calpain plays a role in the modulation of protein synthesis/degradation through the activation of proteasome-dependent proteolysis and inhibition of the mTOR pathway. Severe sepsis significantly increased heart calpain-1 levels and promoted ubiquitin and Pa28β over-expression with a reduction in the mTOR levels. In addition, sepsis reduced the expression of structural proteins dystrophin and β-dystroglycan as well as the contractile proteins actin and myosin. ALLN administration prevented sepsis-induced increases in calpain and ubiquitin levels in the heart, which resulted in decreased of structural and contractile proteins degradation and basal mTOR expression levels were re-established. Our results support the concept that increased calpain concentrations may be part of an important mechanism of sepsis-induced cardiac muscle proteolysis.

## Introduction

The concept of “septic cardiomyopathy” has been described as a complex mechanism resulting in myocardial damage and functional impairment [1–2]. One of possible underlying mechanisms are structural changes associated in the septic hearts, which may contribute to cardiac dysfunction in septic patients [3]. Recently, using an experimental sepsis model based of cecal ligation and puncture (CLP), our research group demonstrated a marked reduction in cardiac dystrophin, an important protein involved in assembling and maintaining of the link between cytoskeletal actin and the extracellular matrix [4]. The presence of heart myofilamental breakdown is characterized by reduced sarcolemmal integrity associated with increased lipid peroxidation and protein nitration in septic hearts causing damage to membrane lipids and cellular proteins [5] that could result in intracellular calcium accumulation [6]. The increased calcium concentration results in activation of calpain, a calcium-dependent intracellular cysteine protease, which results in an upregulation of proteolysis of both target and non-target proteins with consequent irreversible tissue damage [7].

Excessive activation of calpain has been implicated in the pathophysiology of several disorders, including inflammation [8], ischemia-reperfusion [9], trauma [10], diabetes [11], neurodegenerative diseases [12] and myocardial dysfunction [13,14]. Increased activation of calpain results in the subsequent proteolysis of numerous membrane proteins including receptors, growth factors, adhesion molecules and ion transport proteins, enzymes; cytokines and transcription factors [15,16], proteins involved in the link between the cytoskeleton and the plasma membrane (talin, vinculin, spectrin and filamin) and most of the intermediate filament proteins (desmin and vimentin) [17,18]. Importantly, calpain proteolytic activity may eliminate the cross-linking ability of cytoskeletal proteins, especially intermediate filaments, leading to further degradation [7]. Although several cytoskeletal proteins have been identified as substrates, little is known about the molecular mechanisms of protein degradation by calpain. In general, calpains are capable in producing only limited substrate proteolysis resulting in large polypeptide fragments rather than small peptides or amino acids [7]. This limited proteolytic action of calpain has led to speculation that peptide fragments resulting from cleavage are used as substrates for the ubiquitin-proteasome system (UPS) once that system is involved in cellular protein degradation in cardiac myocytes [7,19,20].

UPS has emerged as a key regulator of numerous cellular processes since its initial description over 30 years ago. Composed by a multi-enzymatic cascade that results in the covalent attachment of small molecule ubiquitin to lysine residues within target proteins. The UPS function under normal conditions is essential for the maintenance of protein integrity that make up the sarcomere, mitochondria and the cell membrane including cardiac myocytes, to ensure normal functioning of the heart [20]. Although the UPS has been most commonly studied in the context of cancer biology, increasing evidence suggests that dysfunction of the UPS plays a role in cardiac hypertrophy, ischemia-reperfusion injury and heart failure [21]. However, to date, no study has assessed the role of the ubiquitin-proteasome system in septic cardiomyopathy.

Recent, evidence indicates that calpain plays a dual role in protein metabolism through the concomitant activation of proteasome-dependent proteolysis and inhibition of the PI3K/Akt protein synthesis pathway [22]. Furthermore, mTOR inhibition mediated by calpain could potentially reduce the overall rates of protein synthesis, reducing the available translational machinery required for the maintenance of the protein synthesis levels [15]. Although a regulatory role of the PI3K/Akt/mTOR pathway, partly dependent of calpain, has been demonstrated in cell growth and survival during serum deprivation [22], little has been elucidated about how the PI3K/mTOR signaling pathway is modulated by calpain during cardiac dysfunction.

In rat models of organ dysfunction and circulatory failure caused by endotoxin, treatment with calpain inhibitor has been demonstrated to reduce organ injury and inflammation associated with prevention of circulatory shock and reinstatement of pressor response to noradrenaline [23]. Tissier et al. 2004 demonstrated that endotoxemic rats treated with calpain inhibitor I (ALLN) reduced plasma TNF-alpha and nitrite/nitrate levels and, decreased mesenteric venule leukocyte rolling and adhesion, resulting in improvement of endotoxin-induced cardiac dysfunction [24].

To the best of our knowledge, there are no reports demonstrating a relationship between the inhibition of calpain-1, UPS activation and mTOR inhibition associated with the expression of contractile and cytoskeletal proteins during sepsis-induced myocardial dysfunction. Thus, the aim of this study was to investigate the contribution of calpain-1 to the ubiquitin-proteasome system and mTOR signaling pathway modulation during septic cardiomyopathy induced by cecal ligation and puncture.

## Materials and Methods

### Experimental Animals

Male C57BL/6 mice weighing 22–24 g were maintained at an ambient room temperature (22±2°C) under a 12/12 h light-dark cycle. They were housed at the animal Facility of the Department of Pathology of the Faculty of Medicine of Ribeirão Preto and given standard mouse chow and water *ad libitum*. The animal protocol was approved by the Committee on Animal Research of the Faculty of Medicine of Ribeirão Preto, University of São Paulo, Brazil (Protocol #051/2014). All efforts were made to minimize animal suffering.

### Polymicrobial sepsis model (CLP model)

A modified CLP model was used to induce polymicrobial sepsis. Mice were anesthetized with ketamine (100 mg/kg) and xylazine (10 mg/kg) administered intraperitoneally (i.p.) and prepared for surgery. A midline incision was made in the abdomen and the cecum was exposed and carefully ligated with silk thread below the ileocecal valve. Then, it was punctured with an 18-gauge needle to induce severe septic injury (SSI). The bowel contents were gently extruded through the puncture, and the cecum was then returned to its original position and the abdomen was sutured. Control mice (SHAM) underwent the same procedures, except for cecal ligation and puncture. All mice received subcutaneous doses of saline (50 mL/kg of body weight) immediately and 12 hours after the surgical procedure to prevent dehydration. Tramadol hydrochloride (10 mg/Kg, i.p.) was administered at the end of surgery as well as 12 hours post-surgery for pain relief. Mice were monitored daily for signs of sepsis (12hs/12hs), which typically included piloerection, abnormal gait, slow movements and secretion around eyes. Mice displaying severe signs of distress (labored breathing, non-responsiveness to cage tapping, failure of grooming and severe eye discharge) were humanely sacrificed by injecting a combination of ketamine (90–120 mg/kg) and xylazine (10 mg/kg) followed by cervical dislocation as specified by guide for the care and use of laboratory animals from the Committee on Animal Research of the Faculty of Medicine of Ribeirão Preto, University of São Paulo, Brazil.

### Groups and Treatment

The mice were divided into four groups: 1) SHAM; 2) SHAM+ALLN; 3) SSI; and 4) SSI+ALLN. Half of the mice were administered injections of N-acetyl-leucinyl-leucinyl-norleucinial (ALLN) (i.p., 3 mg/Kg body weight, Sigma-Aldrich Co. St. Louis, MO, USA) diluted in sterile 0.9% NaCl saline (50 μl total volume/animal) four hours after the CLP procedure or sham-operation. Control groups (SHAM and SSI) received an equivalent volume of saline alone.

### Histopathology

For the histopathology analyses, mice were sacrificed by injecting a combination of ketamine (90–120 mg/kg) and xylazine (10 mg/kg) followed by cervical dislocation. After, hearts from SSI, SSI+ALLN, SHAM and SHAM+ALLN groups obtained 24 hours after surgery (n = 6 animals/group) were longitudinally sectioned into two halves, fixed in phosphate-buffered 10% formalin, and embedded in Historesin (Leica Instruments, Heidelberg, Germany) for high-resolution light microscopy, and 2-μm-thick sections were stained with toluidine blue and left ventricles were analyzed.

### Western Blot Analysis

To determine the amount of dystrophin, β-dystroglycan, sarcomeric actin, myosin, ubiquitin, mTOR, calpain-1 and Pa28β in the hearts of septic and SHAM mice, with and without treatment, homogenates of heart left ventricles (n = 6 animals/group) were submitted to immunoblotting 24 hours after the CLP procedure. Fresh hearts left ventricles were washed in cold PBS, and homogenized in extraction buffer supplemented with protease inhibitor cocktail (Sigma-Aldrich, Saint Louis, MO, USA). Total ventricular protein (50 mg protein/well) was resolved on a 5%, 7% or 10% SDS-PAGE (according to protein weight) and transferred to PVDF membranes (Immobilon®-P^sq^, Millipore Corporation, Billerica, MA, USA). The membranes were incubated in blocking solution (5% bovine serum albumin (BSA) in PBS-T) for 24 hours at 4°C with primary antibodies (anti-dystrophin, 1:500, anti-β-dystroglycan, 1:500; Santa Cruz Biotechnology, anti-sarcomeric alpha actin, 1:1500; Sigma-Aldrich, anti-heavy chain cardiac myosin, 1:1000; Abcam, anti-ubiquitin, 1:1000, anti-mTOR, 1:1000, anti-calpain-1, 1:1000, anti-GAPDH, 1:10 000, anti-Pa28β, 1:1000 and anti-α-tubulin, 1:1000; Cell Signaling). Then, the blots were washed and incubated with HRP-conjugated secondary antibodies for 1 hour at room temperature. Membranes were washed, developed using ECL (Amersham Pharmacia Biotech) and visualized using ChemiDoc XRS (BioRad). The image analysis was performed using the public domain *ImageJ Program* (developed at the National Institutes of Health and available at http://rbs.info.nih.gov/nih-image/), using the ‘Gel Analysis’ functions. The analysis results are the values of each band, each of which is proportional to the integrated density value (IDV) of that band, which corresponds to arbitrary units (AU). GAPDH and α-tubulin were used to determine equivalent loading conditions.

### Immunofluorescence

For immunofluorescence studies, left ventricular fragments from SSI and SHAM mice with and without treatment (n = 6 animals/group) were excised 24 hours after surgery and were longitudinally sectioned into two halves, embedded in optimal cutting temperature embedding medium (Tissue-Tek OCT Compound, Sakura Finetek USA, Inc, Torrance, CA, USA), frozen in isopentane chilled in liquid nitrogen, and stored accordingly at -80°C. Immunolabeling was performed (5 mm-thick sections) using primary antibodies to dystrophin (rabbit polyclonal antibody anti-dystrophin, 1:200; Santa Cruz Biotechnology, Santa Cruz, CA, USA). Omission of the primary antibodies served as the negative control.

### RNA isolation, reverse transcription and RT-PCR

Total RNA was isolated from left ventricles (n = 6 animals/group) using TRIzol reagent (Invitrogen, Carlsbad, CA). Quantitative real-time polymerase chain reaction (qPCR) of cDNA with the SYBR II Green QPCR system was performed with glyceraldehyde-3-phosphate dehydrogenase (GAPDH) as the internal control. The nucleotide sequences of the primers from previous studies were as follows: calpain-1 (small subunit-1 (Capns1) (NM_009795)–sense AGCAGGATTCCACCTGAATG e anti-sense–TGGCTCCATAGTGAGCTGTG) and dystrophin (D427) (NM_007868.2)—sense GTTCATTGATGGAGACGGAA and anti-sense–GCACTTCAGCTTCTTCATCT. The reaction products were amplified using the SsoFastTM EvaGreen® Supermix kit (BioRad) with the following cycling conditions: 95°C for 30 seconds, 40 cycles at 95°C for 5 seconds, 60°C for 5 seconds with a final extension at 72°C for 1 minute. The quantification of calpain-1 and dystrophin gene expression was expressed in ΔCT values.

### Statistical Analysis

Data were analyzed using GraphPad Prism 5 statistics program (GraphPad Software Inc., San Diego, CA, USA). Multiple comparisons were performed using a one-way analysis of variance (ANOVA) followed by Tukey (parametric data) or Dunn's (nonparametric data) post hoc multiple-comparisons tests. All data are presented as the mean ± SD. A p<0.05 was considered statistically significant and all *p* values are demonstrated in the graphics.

## Results

### Effect of ALLN administration on cardiac lesions formation

**Fig 1:**
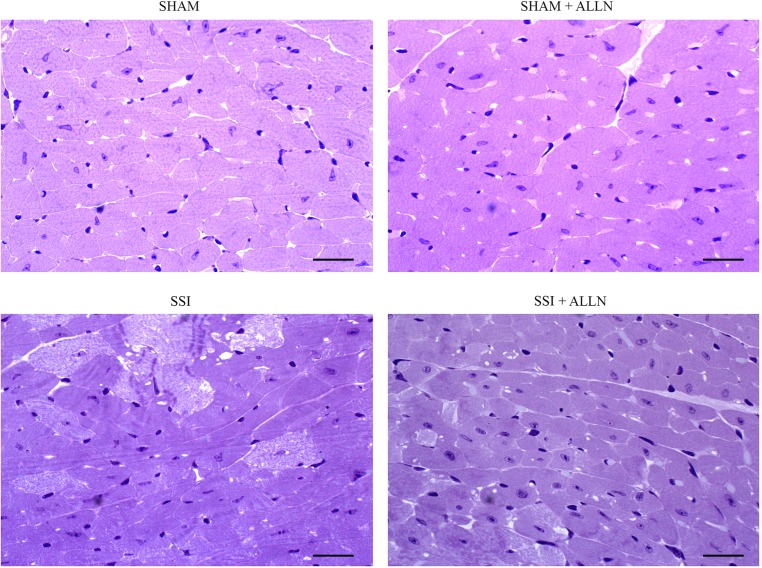
Histopathology of myocardial tissue after CLP. Myocytolysis and contraction bands become evident in the SSI group (upper right panel) compared to the SSI+ALLN group (bottom right panel) 24 h after surgery. The SHAM (upper left panel) and SHAM+ALLN (bottom left panel) groups revealed no morphological alterations. Scale bars indicate 50 mm.

Severe sepsis induced by CLP resulted in intense cellular damage in the myocardium (Fig 1). Cardiac tissue from the SSI group showed widespread and diffuse foci of cardiac myocyte lysis, development of contraction bands necrosis and swollen cytoplasm compared to the hearts of the control animal groups (SHAM and SHAM+ALLN). However, septic mice treated with calpain inhibitor-1 (SSI+ALLN) displayed lower intensity and extended areas of myocytolysis and reduced edema compared with the SSI group at 24 hours after CLP.

### Effect of ALLN administration on calpain-1, ubiquitin and proteasome protein levels and calpain-1 gene expression in the cardiac tissue

**Fig 2:**
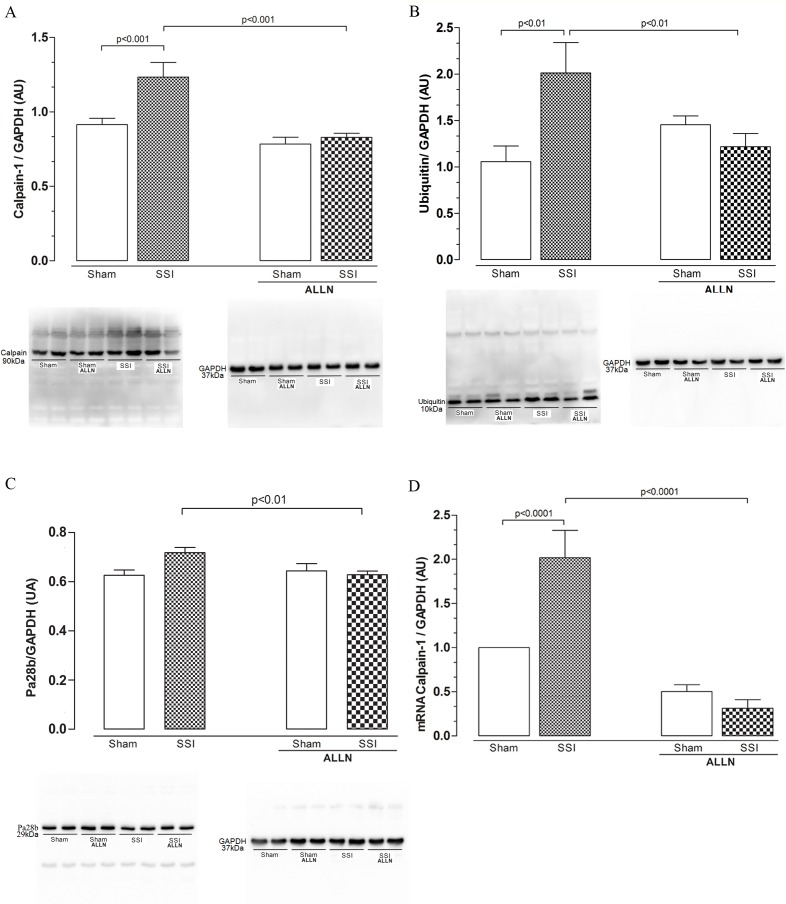
Western blot analysis of cardiac calpain-1, ubiquitin and Pa28β after CLP. Protein levels of calpain-1 (**A**), ubiquitin (**B**) and Pa28β **(C)** in the SHAM, SSI, SHAM+ALLN and SSI+ALLN groups were measured 24 h after the CLP procedure and expressed in arbitrary units (AUs). GAPDH was used to determine equivalent loading conditions. (**D**) Calpain-1 mRNA expression was measured 24 h after the CLP procedure and expressed in arbitrary units (AUs). GAPDH gene expression was used as the internal control for gene expression normalization. The results (n = 6 per group) are representative of three different experiments.

We found that protein levels of calpain-1 in the myocardium of animals receiving CLP (SSI) were increased by approximately 25% compared with SHAM mice (1.23±0.26 vs. 0.91±0.11) 24 hours after the CLP procedure. The SSI+ALLN group demonstrated a marked reduction in calpain-1 levels of approximately 31% compared to the SSI group (0.82±0.06 vs. 1.23±0.26), which exhibited values similar to those of the control groups (Fig 2A). Sepsis increased the ubiquitin protein levels by greater than 901% in the cardiac tissue obtained from the SSI group compared with that of the SHAM group (2.01±0.32 vs. 1.05±0.16) (Fig 2B), whereas in the sepsis group treated with ALLN (SSI+ALLN), the levels of ubiquitin were similar to those of the controls (1.45±0.09 vs. 1.05±0.16). Similarly, the protein levels of Pa28β is increased by approximately 15% in the group (SSI) when compared to the sham group (SHAM) (0.71±0.06 vs. 0.62±0.05), where Pa28β levels remained similar in septic group treated with ALLN (SSI+ALLN) compared to the SHAM values (Fig 2C), suggesting a correlation between increased calpain-1 and ubiquitin-proteasome protein levels during sepsis.

The measurement of cardiac calpain-1 gene expression has been used to examine the effectiveness of ALLN treatment in inhibiting genetic expression of this protease. Non-treated mice submitted to severe sepsis (SSI) displayed increased expression of calpain-1 by 102% when compared with the SHAM group (2.02±0.61 vs. 1.00±0.00). In contrast, the SSI+ALLN group exhibited a significant reduction in calpain-1 gene expression in cardiac tissue as compared to the SSI group (0.50±0.44 vs. 2.02±0.61), as shown in Fig 2D.

### Effects of ALLN administration on cardiac structural and contractile proteins recover

**Fig 3:**
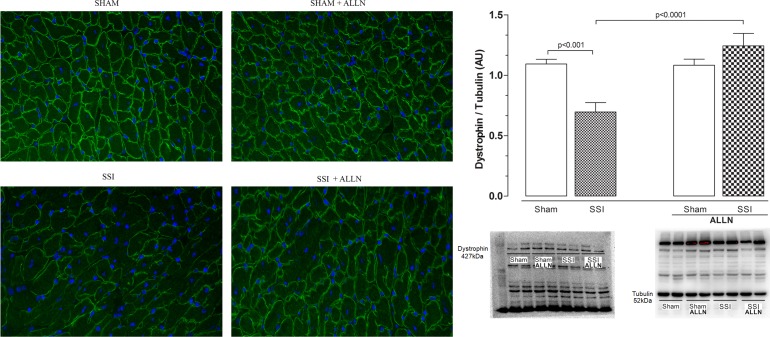
Immunofluorescence and western blot analysis of cardiac dystrophin after CLP. (**A**) Immunofluorescence signal for dystrophin is significantly reduced in the SSI heart (bottom left panel) compared with the immunofluorescent signal in the SHAM heart (upper left panel), and the SHAM+ALLN (upper right panel) and SSI+ALLN (bottom right panel) myocardium. (**B**) Protein levels of dystrophin in the SHAM, SSI, SHAM+ALLN and SSI+ALLN hearts were measured 24 h after the CLP procedure and were expressed in arbitrary units (AUs). α-Tubulin was used to determine equivalent loading conditions. The results (n = 6 per group) are representative of three different experiments. Scale bars indicate 50 μm.

Fig 3 shows that fluorescent signals for dystrophin were markedly reduced in SSI hearts (Fig 3, left bottom panel) compared with SHAM hearts, which exhibited abundant signal for this protein (Fig 3, left upper panel). In contrast, the analysis of the dystrophin fluorescent signal in the SSI group after treatment with ALLN (SSI+ALLN) did not show a significant discrepancy of the fluorescent signal as compared to SHAM or SHAM+ALLN groups (Fig 3, upper panel). In addition, the cardiac levels of dystrophin (Fig 3B) in the SSI group were markedly reduced compared with SHAM mice (0.69±0.19 vs. 1.09±0.07) 24 hours after CLP, representing a reduction of approximately 50%. On the other hand, the treatment of SSI mice with ALLN (SSI+ALLN) significantly prevented the reduction of dystrophin levels in the heart ventricles compared with SHAM or SHAM+ALLN hearts (1.24±0.10 vs. 1.09±0.17 and 1.24±0.10 vs. 1.15±0.12).

**Fig 4:**
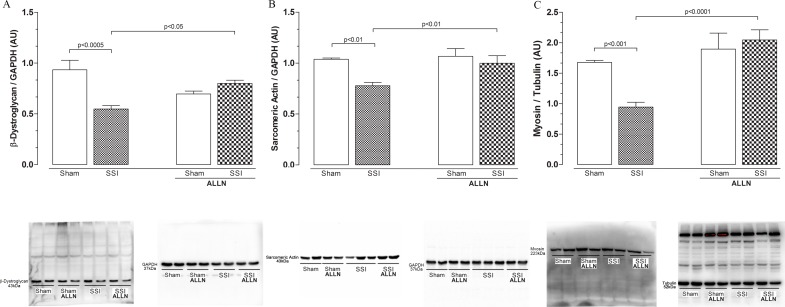
Western blot analysis of cardiac β-dystroglycan, sarcomeric actin and myosin after CLP. Protein levels of β-dystroglycan (**A**), sarcomeric actin (**B**) and myosin (**C**) in the SHAM, SSI, SHAM+ALLN and SSI+ALLN groups were measured 24 h after the CLP procedure and expressed in arbitrary units (AUs). GAPDH was used to determine equivalent loading conditions. The results (n = 6 per group) are representative of three different experiments.

Fig 4 shows the cardiac levels of structural β-dystroglycan and contractile actin and myosin proteins 24 hours after sepsis. The β-dystroglycan levels (Fig 4A) were markedly decreased in SSI hearts compared with SHAM heart ventricles (0.54±0.03 vs. 0.93±0.09), representing a reduction of 40%. In contrast, treatment of SSI mice with ALLN (SSI+ALLN) significantly prevented β-dystroglycan loss compared with the SHAM or SHAM+ALLN hearts (0.80±0.03 vs. 0.93±0.09 and 0.80±0.03 vs. 0.69±0.02).

The contractile proteins, sarcomeric alpha actin and heavy chain cardiac myosin, were reduced by approximately 25% and 48%, respectively, in the SSI hearts compared with the SHAM hearts (Fig 4B and 4C). In contrast, ALLN treatment abrogated the reduction of sarcomeric alpha actin and myosin in the SSI hearts (SSI+ALLN), with values similar to those observed in the sham-treated mice (SHAM+ALLN).

### mTOR inhibition may prevent protein synthesis in sepsis

**Fig 5:**
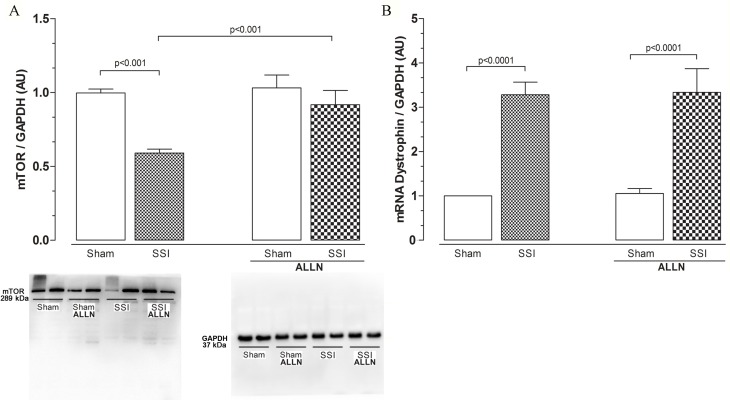
(**A**) Western blot analysis of cardiac mTOR protein levels after CLP. Cardiac mTOR protein levels in the SHAM, SSI, SHAM+ALLN and SSI+ALLN groups were measured 24 h after surgery and were expressed in arbitrary units (AUs). GAPDH was used to determine equivalent loading conditions. (**B**) Analysis of cardiac dystrophin mRNA expression. Dystrophin mRNA expression was measured 24 h after the CLP procedure and expressed in arbitrary units (AUs). GAPDH gene expression was used as the internal control for gene expression normalization. The results (n = 6 per group) are representative of three different experiments.

We then verified mTOR protein levels and dystrophin gene expression. The cardiac levels of mTOR protein (Fig 5A) were reduced by approximately 40% in the SSI mice compared with the SHAM group (0.42 ± 0.05 vs. 0.69 ± 0.06). In contrast, SSI mice treated with ALLN (SSI+ALLN) significantly recovered the expression of mTOR protein levels compared with the SHAM or SHAM+ALLN hearts.

There was an increase in dystrophin gene expression (Fig 5B) in both septic (SSI) and septic mice treated with ALLN (SSI+ALLN) 24 hours after the CLP. This expression represents an increase of approximately 220% for the SSI group compared with the SHAM (3.28 ± 0.69 vs. 1.00 ± 0.00) group and approximately 230% for the SSI+ALLN compared with the SHAM+ALLN (3.33 ± 1.31 vs. 0.93 ± 0.24) group. These alterations in mTOR proteins may explain, in part, the dystrophin gene expression and protein level results.

## Discussion

Herein, we have demonstrated, for the first time, that calpain-1 inhibition reduces the loss of essential cardiac cytoskeletal (dystrophin and β-dystroglycan) and contractile (sarcomeric actin and myosin) proteins during sepsis. These changes were accompanied by the inhibition of ubiquitin, a protein implicated in the degradation of intracellular proteins, and Pa28β, a protein that participates of proteasome activity. We also observed increased mTOR protein expression, a key component of the PI3K/Akt/mTOR signaling pathway involved in cell survival and proliferation. These results indicate its importance as a possible regulator of protein turnover in sepsis-induced cardiac dysfunction.

Calpain activation plays an important role in the mechanisms of many acute inflammatory disease of the cardiovascular system [23,24]. Previous studies have shown the peak of calpain activity approximately 6 hours after sepsis induction and that calpain activation may play an important role in the course of sepsis and multiple organ failure [25]. Our results demonstrated an increase of approximately 25% of calpain-1 levels in septic hearts, 24 hours after CLP. On the other hand, the cardiac calpain-1 levels were markedly reduced in septic-treated hearts indicating the effectiveness of ALLN treatment in the inhibition of both protein and genetic expression of this protease.

In cardiac muscle, dystrophin is part of a glycoprotein complex that works to provide muscle tensile strength and protect fibers from damage during muscle contraction and relaxation. Disruptions in the dystrophin-glycoprotein complex are associated with decreased stability and integrity of the sarcolemma. This weakens the connection between the structural framework of cells (cytoskeleton) with the lattice of proteins and the extracellular matrix [26–29] resulting in myocardial dysfunction and heart failure [30].

Cardiac myocytes of dystrophin-deficient mdx mice are abnormally vulnerable to mechanical stress and sarcolemmal injury accompanied by decreased systolic and diastolic left ventricular dysfunction [31], suggesting that dystrophin disruption could be associated with the progression of cardiomyopathy as a result of mechanical stress and damage induced by the overload [32,33]. In the present study, we demonstrate that severe sepsis induced a significant reduction in cardiac structural proteins dystrophin and β-dystroglycan examined 24 hours after induction of sepsis employing the CLP model. In addition, decreased amounts of contractile proteins, sarcomeric actin and myosin, in cardiac myocytes were followed by significant increases in calpain cardiac muscle levels. Treatment of septic mice with the calpain inhibitor-1 ALLN prevented the loss/reduction of the cardiac proteins.

The contribution of calpain to the proteolysis of cardiac proteins is of interest since calpain cannot completely degrade some proteins such as actin and myosin [7,34,35]. In this pathway, the UPS would be responsible for the achievement of proteolytic action initiated by calpain in situations of cellular catabolism [36]. One of the central components of proteolytic pathway of both nuclear and cytosolic compartments is the 20S proteasome since its action is considered a prerequisite for effective action of the ubiquitin pathway [37]. Interestingly, the structure of 20S proteasome presents 28 subunits and can be divided in α and β families [38,39]. The β subunits show a higher degree of sequence diversity than α subunits. Almost all β subunits initially possess an N-terminal pro sequence, which is cleaved off during particle formation. This step exposes, on most β subunits, a terminal threonine residue that is necessary for activity and several observations indicate that the β subunits are responsible for the proteolytic activities [40–42]. There are few data in the literature showing an association between calpain-1 and the UPS in cardiac dysfunction during sepsis, and there are no reports evaluating this relationship with structural and contractile protein expression in septic myocardium. Our results show increased levels of ubiquitin, Pa28β and calpain-1 combined with reduced amounts of structural and contractile proteins in the hearts of septic mice. These observations demonstrate a combined action of these two proteolytic pathways in septic myocardium. In addition, the inhibition of calpain-1 confirmed the importance of this protease in the development of septic cardiomyopathy.

A previous study using neonatal cardiac myocytes after transfection with adenovirus demonstrated an increased calpain-1 expression associated with proteolysis of troponin I, desmin and protein kinase Cα, without apoptosis. In addition, the transfection with adenovirus caused augmented levels of ubiquitinated proteins, which were inhibited by the co-expression of an endogenous inhibitor of calpain, the calpastatin [43]. The evaluation of cardiac overexpression of calpain-1 in transgenic mice showed high levels of protein ubiquitination and proteasomal activity with functional cardiac changes such as the reduction of the ejection fraction and dilatation of the cardiac chambers associated with lethality [44].

Analysis of dystrophin gene expression demonstrated the over-expression of dystrophin mRNA in the hearts of septic mice treated and not treated with ALLN, which was not consistent with the results found for the dystrophin protein levels assessed by immunoblotting and fluorescence. Herein, the results suggest reasonable alterations in protein synthesis in the heart of mice subjected to experimental sepsis. Based on these results, it has become essential to assess the mTOR protein levels involved in the control of protein synthesis, metabolism and cell cycle in an attempt to correlate these findings with calpain-1 activation during sepsis.

Recently, it has been suggested that calpain is capable of partially modulating cellular proliferative signals dependent on the PI3K/mTOR signaling pathway [22]. In cases of cardiac disease, where there is no effective process of neo-vascularization, repair and tissue regeneration may be limited or may not occur. Another important aspect is that during ischemic cardiac injury, the activation of the mTOR pathway may play a protective role against oxidant injury caused by ischemia/reperfusion in cardiac myocytes [45]. This occurs because during periods of post-ischemic injury, mTOR is necessary for preventing apoptosis in cardiac cells [45]. In studies that involve blocking the phosphorylation and activation of p70S6K, a component of the mTOR signaling pathway, the cardiac ischemic and scar areas are increased, demonstrating a cardioprotective role for mTOR [46]. In the present study, the protein levels of mTOR were reduced in the myocardium of septic mice, which could explain, at least in part, the unrecovered/reduced protein levels of dystrophin even with high mRNA expression. These results suggest that the decreased levels of mTOR could be associated with reduced protein synthesis in the myocardium of septic mice, which did not occur in animals treated with ALLN. In addition, our results demonstrate that calpain-1 also plays a key role in protein breakdown under septic conditions, which would act as a mechanism of protein synthesis disruption, thus increasing the degree of cardiac dysfunction and injury due to sepsis.

## Conclusions

In conclusion, our data provide an important insight into the role of calpain-1 in the development of septic cardiomyopathy because its inhibition reduced/mitigated the loss of key cardiac proteins. Furthermore, our results demonstrate a significant connection between two proteolytic pathways: calpain and the ubiquitin-proteasome system. Finally, breakdown of structural and contractile proteins would be associated with a possible reduction in protein synthesis through the inhibition of mTOR protein. These findings provide further important information about the use of a calpain inhibitor as a possible strategy to prevent the progression to heart failure in sepsis.
